# Digital Therapeutic (Mika) Targeting Distress in Patients With Cancer: Results From a Nationwide Waitlist Randomized Controlled Trial

**DOI:** 10.2196/51949

**Published:** 2024-04-25

**Authors:** Franziska Springer, Ayline Maier, Michael Friedrich, Jan Simon Raue, Gandolf Finke, Florian Lordick, Guy Montgomery, Peter Esser, Hannah Brock, Anja Mehnert-Theuerkauf

**Affiliations:** 1 Department of Medical Psychology and Medical Sociology, Comprehensive Cancer Center Central Germany, University Medical Center Leipzig Leipzig Germany; 2 Fosanis GmbH Berlin Germany; 3 Department of Medicine II University Medical Center Leipzig Leipzig Germany; 4 University Cancer Center Leipzig Comprehensive Cancer Center Central Germany Leipzig Germany; 5 Center for Behavioral Oncology, Department of Population Health Science and Policy, Icahn School of Medicine at Mount Sinai New York, NY United States

**Keywords:** digital therapeutic, digital health, mobile health, app, cancer, randomized controlled trial, supportive care, oncology, access to care, distress, depression, anxiety, fatigue, mobile phone

## Abstract

**Background:**

Distress is highly prevalent among patients with cancer, but supportive care needs often go unmet. Digital therapeutics hold the potential to overcome barriers in cancer care and improve health outcomes.

**Objective:**

This study conducted a randomized controlled trial to investigate the efficacy of Mika, an app-based digital therapeutic designed to reduce distress across the cancer trajectory.

**Methods:**

This nationwide waitlist randomized controlled trial in Germany enrolled patients with cancer across all tumor entities diagnosed within the last 5 years. Participants were randomized into the intervention (Mika plus usual care) and control (usual care alone) groups. The participants completed web-based assessments at baseline and at 2, 6, and 12 weeks. The primary outcome was the change in distress from baseline to week 12, as measured by the National Comprehensive Cancer Network Distress Thermometer. Secondary outcomes included depression, anxiety (Hospital Anxiety and Depression Scale), fatigue (Functional Assessment of Chronic Illness Therapy-Fatigue), and quality of life (Clinical Global Impression-Improvement Scale). Intention-to-treat and per-protocol analyses were performed. Analyses of covariance were used to test for outcome changes over time between the groups, controlling for baseline.

**Results:**

A total of 218 patients (intervention: n=99 and control: n=119) were included in the intention-to-treat analysis. Compared with the control group, the intervention group reported greater reductions in distress (*P*=.03; ηp²=0.02), depression (*P<*.001; ηp²=0.07), anxiety (*P=*.03; ηp²=0.02), and fatigue (*P=*.04; ηp²=0.02). Per-protocol analyses revealed more pronounced treatment effects, with the exception of fatigue. No group difference was found for quality of life.

**Conclusions:**

Mika effectively diminished distress in patients with cancer. As a digital therapeutic solution, Mika offers accessible, tailored psychosocial and self-management support to address the unmet needs in cancer care.

**Trial Registration:**

German Clinical Trials Register (DRKS) DRKS00026038; https://drks.de/search/en/trial/DRKS00026038

## Introduction

### Background

In addition to somatic symptoms such as pain [1], patients with cancer report elevated levels of distress, anxiety, and depression [2,3]. Epidemiological data show that the prevalence of clinically substantial psychological distress typically ranges from 30% to 60% among patients with cancer [2,4]. Psychological distress can persist long after the end of treatment and is associated with reduced quality of life (QoL), lower cancer treatment adherence, and lower survival rates [5].

Supportive care interventions to prevent and manage the adverse psychological and physical effects of cancer across the cancer trajectory effectively improve outcomes such as emotional distress, QoL, and fatigue [6]. Optimal supportive care is holistic and patient centered, that is, based on the needs of each individual patient [7]. However, access to supportive care is often limited by a lack of specialist staff, organizational deficiencies, and barriers that cause patients to avoid or delay their treatment [8-10]. Thus, emerging or persistent supportive care needs across the cancer trajectory often go unmet, with detrimental psychosocial and emotional impacts on patients with cancer [11]. Moreover, the number of patients living with cancer has increased rapidly in recent years [12] due to improved early detection, diagnosis, and oncological treatments, posing a growing challenge to health systems worldwide to ensure adequate and long-term care for all patients with cancer [13].

The increasing use of digital health has ushered in a new era of patient-centered cancer care due to its potential for cancer care delivery [14]. Digital health interventions provide multiple benefits: they facilitate easy and low-threshold access to care, can overcome barriers to care (eg, location, time, and health status), may enhance symptom management through real-time symptom assessment, are scalable, and provide cost-effective and efficient information sharing [14]. Growing literature suggests that digital therapeutics, a subset of digital health interventions providing evidence-based treatments driven by software, play a useful role in addressing the unmet needs of patients with cancer [15]. For instance, various mobile apps have proven to be effective in catering to specific needs of patients with cancer, such as pain, anxiety, or QoL, by using different types of interventions, such as psychoeducation, physical exercises, or coping skills training (eg, [16-19]). Moreover, large analyses such as systematic reviews and meta-analyses evaluating the efficacy of app-based interventions for patients with cancer show positive effects on patient-relevant outcomes, such as distress, QoL, anxiety, depression, pain, and fatigue [20-23].

Existing app-based supportive care interventions provide various intervention modules, such as symptom monitoring, psychoeducation, mindfulness exercises, physical exercises, and cognitive behavioral therapy (CBT) techniques [24]. However, most of these apps are limited in their scope, targeting only specific symptoms (eg, fatigue) [25] and health behaviors (eg, physical activity) [26], or provide only a single function (eg, mindfulness training or symptom tracking) [27-29]. Furthermore, some of these apps were originally developed for non–oncology patient populations and have only been slightly adapted for patients with cancer [30]. Only a few apps offer a broader range of intervention modules [25,31], but they target specific subgroups of patients with cancer (eg, patients with 1 tumor entity or with specific symptoms).

Despite the evident need, there is yet no digital therapeutic that comprehensively addresses the problems faced by all patients with cancer and simultaneously offers tailored support for each individual patient. Therefore, we investigated the efficacy of Mika (developed by Fosanis GmbH), an app-based digital therapeutic that addresses all patients with cancer transdiagnostically and provides a holistic supportive care intervention. The app incorporates evidence-based supportive care elements, such as distress and symptom monitoring [32], CBT-based coping skills training [33], mindfulness-based stress reduction (MBSR) [34,35], strength and flexibility training [36], and patient education [37], thus targeting different aspects of psychological distress. An artificial intelligence algorithm individually tailors the content of the app to patients’ needs, considering cancer type, cancer treatment stage, and use behavior. A previously conducted pilot study of 70 patients with gynecological cancer indicated Mika’s feasibility and potential efficacy [38]. Considering the significant prevalence and impact of psychological distress among patients with cancer, this condition was selected as the primary end point of our study. This is underscored by the app’s integrated features for distress tracking and management alongside the widespread recommendation for distress screening in routine clinical care. Distress is recognized as a crucial clinical marker for assessing the efficacy of interventions across various tumor types and catering to the immediate and long-term supportive care needs of this patient group.

### Objectives

The primary aim of this waitlist randomized controlled trial (RCT) was to examine the efficacy of the Mika app for general distress in patients with cancer. The secondary aim was to assess the efficacy of the Mika app on anxiety, depression, fatigue, and QoL. We hypothesized that participants receiving access to the Mika app plus usual care (UC) for 12 weeks would report greater reductions in distress, anxiety, depression, and fatigue and greater improvements in QoL compared to participants receiving UC only.

## Methods

### Study Design

This nationwide unblinded 2-arm waitlist RCT evaluated the efficacy of the app-based digital therapeutic Mika in reducing distress in patients with cancer and was conducted fully decentralized in Germany, that is, participant recruitment, delivery of the study intervention, and outcome data collection were conducted without involving in-person contact between the study team and the participants. In this RCT, participants were assigned to either (1) access to the Mika app plus UC (intervention group [IG]), or (2) UC alone (control group [CG]). Participants were assessed at baseline (t0), 2 weeks (t1), 6 weeks (t2), and 12 weeks (t3) using self-report questionnaires. Once the participants in the CG completed the 12-week questionnaire, they also received access to the Mika app.

### Ethical Considerations

The trial was approved by the Ethics Committee of the Medical Faculty of Leipzig University (404/21-ek) and was registered at the German Clinical Trials Register (DRKS00026038) in October 2021. All participants provided written informed consent prior to their participation in the study and retained the autonomy to withdraw from the study at any time. All personal data collected and used for this study underwent deidentification to safeguard the anonymity of participants. Monetary compensation was not provided to participants for their involvement in the study.

### Participants

Textbox 1 shows the inclusion and exclusion criteria for this study. We only included patients who had been diagnosed with cancer or relapse within the last 5 years as they are likely to feel burdened by the physical and psychological effects of the disease and its treatment and therefore require supportive care. Epidemiological data indicate that supportive care needs typically decline in the years of long-term survivorship (cancer or relapse diagnosis ≥5 years ago) [39]. Participants were required to confirm their cancer diagnosis during the course of the study by submitting a letter from their treating physician. The study team enrolled patients after they had provided written informed consent, which had to be completed at home and submitted by email or mail.

**Inclusion and exclusion criteria for this study.**

**Inclusion criteria**

- Age≥18 years
- Cancer diagnosis or relapse diagnosis within the last 5 years (10th revision of the International Statistical Classification of Diseases and Related Health Problems: C00-C97)
- Access to a smartphone or tablet
- Ability to provide informed consent

**Exclusion criteria**

- Insufficient German language skills
- Inability to use a smartphone or tablet
- Prior use of the investigated digital therapeutic

### Random Assignment

Participants were randomly assigned (1:1) to either the IG or CG using permuted block randomization with blocks of 4 based on an a priori created randomization list. The allocation sequence was concealed from the study investigators until assignment. Due to the nature of the intervention, it was not feasible to blind participants or the study team to the group assignment.

### Recruitment and Procedure

Between September and November 2021, patients were recruited via social media advertising campaigns (Facebook and Instagram, Meta Inc) and informational emails to cancer support groups that directed patients to the trial website with a contact form for study registration. In addition, patients were recruited from a participant pool consisting of participants from previous independent studies at the University Medical Center Leipzig. Patients from the participant pool were approached directly by the study team via phone.

All interested patients were screened by phone to determine eligibility. To identify patients who were already users of the digital therapeutic, the study team asked participants about their use of digital support, however, without referring to the publicly available digital therapeutic by name to prevent CG patients from accessing the digital therapeutic before their enrollment in the study. Eligible patients received study information in the form of a video and text via email. Patients were informed that they were required to submit a physician’s letter confirming their cancer diagnosis via a secure cloud data-sharing service (TeamDrive, Crunchbase) during the course of their study participation. After providing informed consent, the participants were randomized into the IG or CG and completed the baseline questionnaires. Participants were informed about their group assignment following a completed baseline assessment. IG participants received a study access code to activate the app after downloading it from the app stores for either Android or iOS smartphones, allowing free use. The questionnaire battery was administered electronically using LimeSurvey (LimeSurvey GmbH). All participants received email invitations and reminders at 2, 6, and 12 weeks to complete the questionnaire. This RCT focused on changes in outcomes from baseline (t0) to week 12 (t3). The 2 assessments in between (t1 and t2) were not part of the analysis; an analysis of the trajectory of the symptoms is planned for the future. Once the CG participants completed the 12-week questionnaire, they also received a study access code that could be used to activate the app. All the participants received information about the app’s content and technical application via a standardized telephone introduction to the app. All participants were contacted for an exploratively structured telephone interview after completing the 12-week questionnaire. During this interview, the use of psychotherapeutic support during study participation was assessed. Data collection ended in March 2022.

### Monitoring

Data monitoring was performed via standardized phone calls following questionnaire completion of each participant across all measurement time points to ensure data validity. These phone calls served to ask participants to provide missing questionnaire data, to allow participants to clarify difficulties in understanding single questionnaire items, and to provide assistance with limited app functionality. Missing questionnaire data were entered directly into the database by the study team, with a study team member reading the unanswered questions and associated response options to participants verbatim, prompting them to select their response option.

Self-reported adverse reactions and side effects of the investigated digital therapeutic were assessed at each measurement time point as part of the web-based questionnaire battery.

### Intervention

**Figure 1:**
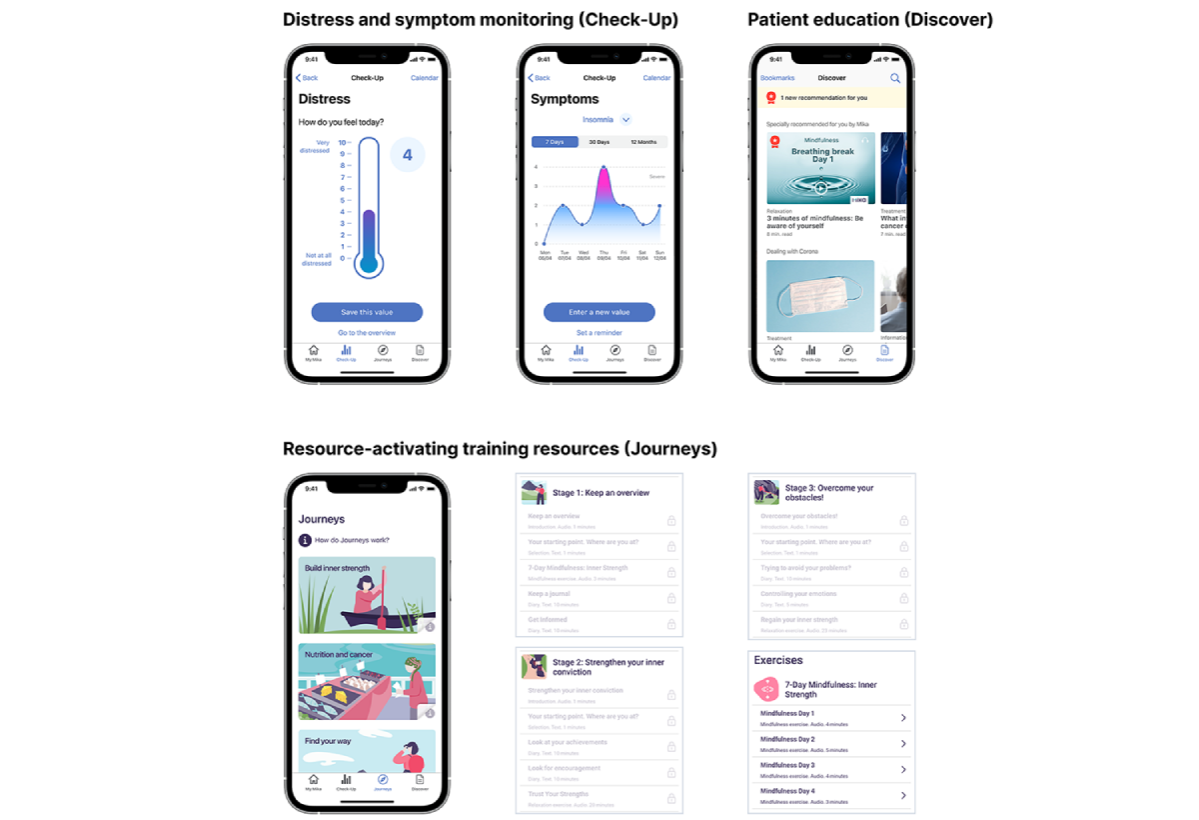
Screenshots of the Mika app modules.

Mika is an app-based digital therapeutic that provides a personalized supportive intervention aiming to reduce distress associated with cancer and its medical treatment, thus improving patients’ QoL. Mika comprises 3 modules: *Check-Up*, *Discover,* and *Journeys*. The *Check-Up* module allows for the monitoring of distress and symptom monitoring with electronic patient-reported outcomes that can be shared and discussed with the attending physician. The *Discover* module delivers coaching via articles and videos on cancer types and medical treatments, psychological well-being, physical activity, diet, and social and financial issues, which are based on scientific evidence and presented in a clear and understandable manner for patients. The *Journeys* module provides users with evidence-based, resource-activating training courses combining psychoeducation and exercises to help patients cope with the mental and physical effects of cancer, for example, coping with stress and fatigue, making decisions, or living with immunotherapy (for more details on the app modules, refer to Table 1 and Figure 1). An artificial intelligence algorithm within the app customizes the content for each patient. This includes personalized recommendations based on cancer type, cancer treatment stage, and crucially; the nature and severity of reported symptoms; and ensuring personalized support for each individual. This customization process not only accounts for general patient information but also actively incorporates real-time symptom tracking data and user reading behavior using an attentional factorization machine that predicts a patient’s likelihood of engaging with specific content. This approach focuses on important feature interactions related to content consumption [40], ensuring that recommendations are dynamically adjusted as patients report changes in symptoms and interact with the content. In addition, the algorithm uses a Dirichlet loss function to estimate the uncertainty in predictions [41], allowing the content to be ranked and presented based on the estimated read probability. The model undergoes monthly updates using historical data, optimizing through hyperparameter tuning evaluated by 7-fold time series cross-validation.

It is hypothesized that the digital therapeutic empowers patients with cancer by improving their health literacy and self-management along the cancer trajectory using evidence-based methods, such as symptom monitoring, patient education, MBSR, strength and flexibility training, acceptance and commitment therapy, and CBT-based coping skills training.

The Mika app was developed by Fosanis GmbH in collaboration with leading research institutions, such as the Charité University Hospital Berlin, University Hospital Leipzig, and the National Center for Tumor Diseases Heidelberg. All content of the app was carefully reviewed by experts (eg, oncologists, psychotherapists, nutritionists, and physiotherapists) before publication. The feasibility and preliminary efficacy of Mika were investigated in a previously conducted randomized pilot study involving 70 patients with gynecological cancer [38]. Mika is available for download free of charge in German and the United Kingdom app stores for Android and iOS smartphones.

IG participants could freely choose the modules to work on. While regular app use was recommended, participants were instructed to use the app at least 3 times a week.

**Table 1:** Mika app modules and description.

Distress and symptom monitoring (Check-Up)	Resource-activating training courses (Journeys)	Patient education (Discover)
Monitoring tools (electronic PROs^a^) allow for continuous monitoring of distress levels and symptoms using diary and calendar features. Wearables can be connected to complement PROs. Tracked data can be exported and shared with the treatment team (format: human-readable PDF, machine-readable Fast Healthcare Interoperability Resources). **Monitoring of distress levels** Distress levels or areas can be tracked using the National Comprehensive Cancer Network Distress Thermometer (scale range 0-10) and problem list (practical problems, family problems, emotional problems, spiritual or religious concerns, and physical problems). A value exceeding the threshold of ≥5 prompts a warning that psychosocial support might be indicated. **Monitoring of symptoms** Patients can choose from a list of 58 common symptoms along the cancer continuum and can add additional individual symptoms. Severity of symptoms are documented using PRO Common Terminology Criteria for Adverse Events items. If tracked symptom severity values exceed the predefined scale thresholds, a warning is triggered advising the patient to seek medical consultation.	Treatment and recovery programs combining patient education and exercises (journaling, mindfulness, relaxation, physical activity, and infotainment) to help patients cope with the mental and physical effects of cancer. The several days courses are designed to strengthen patients’ resilience and trigger behavioral change. **List of available Journeys** Stress relief Gain control Find your way Sources of strength Control your feelings Accept your body Alleviate fatigue Making decisions Living with immunotherapy Maintenance therapy with Zejula Ovarian cancer treatment Living with breast cancer Yoga and cancer Nutrition and cancer	Magazine-like content hub consisting of educational articles and videos addressing psychological well-being, physical activity, diet, and social and financial issues. Topics are structured into different categories: Cancer and treatment types Symptoms and side effects Nutrition in cancer Healthy lifestyle Partnership and family Relaxation Exercise training Law and finances COVID-19 and cancer Law and finances Recipes Survivor stories

^a^PRO: patient-reported outcome.

### UC Condition

UC consisted of all health care that patients in Germany usually receive. There were no restrictions on health care use.

### Outcome Assessment

#### Primary Outcome

The primary outcome was the change in psychological distress from baseline to 12 weeks, measured using the validated German version of the National Comprehensive Cancer Network Distress Thermometer [42]. Distress Thermometer is a well-established single-item self-report measure that assesses the global level of distress on a 0 (no distress) to 10 (extreme distress)-point Likert scale. It shows excellent psychometric properties across various cancer populations worldwide and is recommended as a clinical tool for routine clinical care [43]. A score ≥5 indicates clinically significant levels of distress.

#### Secondary Outcomes

The secondary outcomes included changes in anxiety and depression symptoms, fatigue from baseline to 12 weeks, and QoL at 12 weeks. Anxiety and depression symptoms were measured using the Hospital Anxiety and Depression Scale [44], a 14-item self-report measure of anxiety and depression, with 7 items measuring each subscale. Scores for each subscale range from 0 to 21, with a higher score indicating higher levels of anxiety or depression and a cutoff score of ≥8 for each subscale. Fatigue was assessed using the Functional Assessment of Chronic Illness Therapy-Fatigue [45], a 13-item measure that assesses self-reported tiredness, weakness, and difficulty in performing usual activities due to fatigue. The Functional Assessment of Chronic Illness Therapy-Fatigue score ranges from 0 to 52, with higher scores representing less fatigue. Self-reported QoL was measured using an adapted version of the Clinical Global Impression-Improvement Scale [46], a single-item 7-point measure that assesses the overall improvement of a patient’s disease relative to a baseline state at the beginning of the intervention. In this trial, the Clinical Global Impression-Improvement Scale measured improvement in QoL relative to the beginning of the study, with a value of 4 indicating no change, <4 indicating improvement, and >4 indicating deterioration in QoL.

### Intervention Safety

The safety of the digital therapeutic was assessed by the number and type of self-reported adverse reactions and side effects during the trial duration.

### Intervention Adherence and Engagement

Adherence to the intervention was assessed by tracking app activities. IG participants were considered active once they activated the app using the study access code and consented to the Mika app’s privacy terms. Subsequently, their pseudonymized in-app activities were automatically recorded as log data. These log data facilitated the evaluation of intervention adherence, defined as the number of days with ≥1 app activity during each of the three 4-week periods (0-4, 5-8, and 9-12 weeks) within the 12-week intervention. Such an approach enabled us to capture the frequency and diversity of app engagement, thus embodying a comprehensive definition of adherence. In addition, engagement across the app’s 3 modules—Check-Up, Discover, and Journeys—was analyzed.

### Statistical Analysis

Given an estimated dropout rate of 20% (50/250), a priori sample calculations showed that a sample of 2×125 (N=250) at baseline was needed to detect a change of 1 scale point (SD 2; α=.05; 1−β=.8) in the primary outcome.

Primary analyses were performed using the intention-to-treat (ITT) principle, which included all randomized participants with a confirmed cancer diagnosis by a physician’s letter. Analyses were also performed per-protocol (PP), which was restricted to participants who (1) completed the self-report questionnaire at all measurement time points, (2) did not receive psychotherapeutic support during study participation, (3) did not use the investigated digital therapeutic before receiving access during study participation, and (4) used the investigated digital therapeutic at least 1 time per period up to the 5- to 8-week period of the 12-week intervention period (only IG).

Analysis of covariance was used to examine changes in distress, depression, anxiety, and fatigue outcomes between the trial arms from baseline to 12 weeks, controlling for baseline scores. Exploratory regression analyses were conducted to investigate potential variables influencing the primary outcome. These analyses focused exclusively on sociodemographic and clinical factors that showed differences between the IG and CG in the initial group comparison. Partial eta–squared was reported as the effect size for all analyses of covariance, with effect sizes interpreted as small, medium, and large at ≥0.01, ≥0.06, and ≥0.14 [47], respectively. Differences in QoL between trial arms at follow-up (12 weeks) were analyzed with a 2-tailed 2-sample *t* test, using Hedges *g*' as a measure of effect size (≥0.2=small effect, ≥0.5=medium effect, and ≥0.8=large effect [47]).

Missing outcome data at random were imputed using the expectation-maximization algorithm. For dropouts, the last observation carried forward was used. For deceased participants, the worst possible values were assumed. Dropouts were participants who failed to complete the baseline or follow-up questionnaires or failed to provide a physician’s letter confirming their cancer diagnosis. A dropout analysis was performed to compare the variables of age, sex, and baseline distress between study noncompleters (dropouts) and study completers using chi-square and *t* tests. Furthermore, to model the robustness of the primary efficacy analysis under different assumptions for missing data mechanisms, an explorative sensitivity analysis using reference-based multiple imputation (jump-to-reference) [48] was performed in the extended ITT population (all randomized participants). For this purpose, monotone missing values were replaced using the jump-to-reference approach, whereas sporadic missing values were replaced under the assumption of missing at random. For jump-to-control and jump-to-reference imputation, 50 data sets were generated to minimize the loss of statistical power. The results were then aggregated across the imputed data sets [49].

All statistical tests were 2-tailed, with a significance level of 5%. Analyses were performed using R (version 4.1.0; R Foundation for Statistical Computing) [50].

## Results

### Study Sample

**Figure 2:**
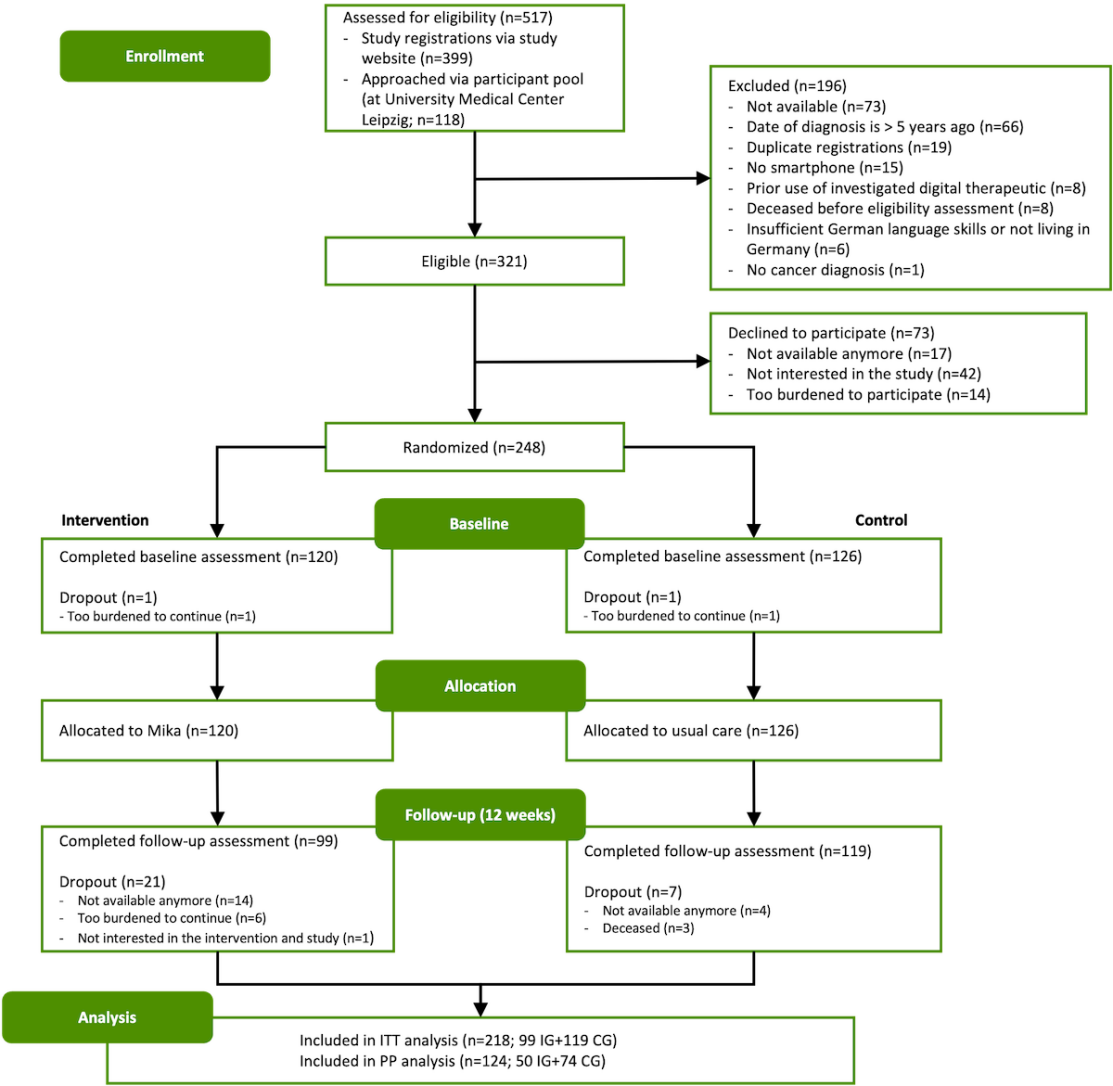
CONSORT (Consolidated Standards of Reporting Trials) diagram. CG: control group; IG: intervention group; ITT: intention-to-treat; PP: per-protocol.

Over the 3-month recruitment period, 517 persons were screened for eligibility and 321 were determined eligible. Of the 321 participants, 248 (77.3%) gave informed consent and were randomly assigned to the IG and the CG (Figure 2). Of the 248 participants, 37 (14.9%) were considered dropouts because they did not complete baseline or follow-up assessments (n=7), failed to confirm their cancer diagnosis by submission of a physician’s letter (n=7), or both (n=23). Age and sex of study dropouts and study completers did not differ (*P_age_*=.89 and *P_sex_*=.23), but participants who dropped out showed higher distress levels at baseline compared to study completers (*P*=.02). Participants without a verified cancer diagnosis (30/248, 12.1%) were excluded from the ITT analysis, resulting in an ITT population of 218 participants (n=99, 45.4% IG and n=119, 54.6% CG). Of the 218 participants, 173 (79%) were recruited via social media advertisements and cancer support groups and 45 (21%) were recruited using the participant pool of prior studies.

Baseline characteristics were balanced between the groups (Table 2), but participants in the IG were younger compared with those in the CG (*P*=.02). No baseline differences in the primary and secondary outcome parameters were observed between the groups, with *P* values as follows: *P*=.99 (distress), *P*=.25 (depression), *P*=.47 (anxiety), and *P*=.21 (fatigue). On average, participants were 56 (SD 11) years old, and 60.6% (132/218) of the participants were female and had been diagnosed with cancer 25 (SD 17) months earlier. The most frequently reported cancer types were breast cancer (74/218, 33.9%) and hematological cancer (61/218, 28%), with 8.7% (19/218) of participants reporting a diagnosis of relapsed cancer. The PP population comprised 124 participants, following the exclusion of 94 participants. The primary reasons for exclusion were psychotherapeutic support during study participation and prior use of the investigated digital therapeutic.

**Table 2:** Baseline sociodemographic and medical sample characteristics (n=218).

Variable			Intervention^a^ (n=99)		Control^b^ (n=119)
Age (years), mean (SD; range)			55 (11; 20-77)		58 (10; 35-76)
**Sex, n (%)**					
	Female	65 (65.7)		67 (56.3)	
	Male	34 (34.3)		52 (43.7)	
**Cancer type, n (%)**					
	Breast	38 (38.4)		36 (30.3)	
	Hematological	27 (27.3)		34 (28.6)	
	Lung	9 (9.1)		12 (10.1)	
	Urogenital	6 (6.1)		14 (11.8)	
	Gastrointestinal	7 (7.1)		11 (9.2)	
	Head-neck	3 (3)		4 (3.4)	
	Gynecologic	3 (3.0)		3 (2.5)	
	Other	6 (6.1)		5 (4.2)	
Relapse, n (%)			7 (7.1)		12 (10.1)
Time (months) since cancer diagnosis, mean (SD)			25 (18)		24 (16)
Psychotherapeutic support during study participation, n (%)			15 (15.2)		17 (14.3)
Digital therapeutic use before study, n (%)			15 (15.2)		14 (11.8)
NCCN Distress Thermometer≥5^c^, n (%)			71 (71.8)		86 (72.3)
Anxiety (HADS-A)≥8^d^, n (%)			56 (56.7)		67 (56.3)
Depression (HADS-D)≥8^e^, n (%)			42 (42.4)		41 (34.5)
**Exclusion in per-protocol analysis^f^, n (%)**			49 (49.5)		45 (40.9)
	Psychotherapeutic support during study	15 (15.2)		17 (14.3)	
	Prior use of investigated digital therapeutic	15 (15.2)		28 (23.5)	
	Missing questionnaire	5 (5.1)		5 (4.2)	
	Digital therapeutic was not used up until the 5- to 9-week period	17 (17.2)		N/A	

^a^Intervention=12-week access to digital therapeutic app intervention+usual care.

^b^Control=usual care.

^c^NCCN Distress Thermometer: National Comprehensive Cancer Network Distress Thermometer (at baseline, clinically significant level of distress≥5).

^d^HADS-A: Hospital Anxiety and Depression Scale, anxiety subscale (German version, at baseline, cutoff score ≥8).

^e^HADS-D: Hospital Anxiety and Depression Scale, depression subscale (German version, at baseline, cutoff score ≥8).

^f^Multiple reasons are possible within 1 patient, and cases do not add up to the total number.

### Primary Outcome

After 12 weeks, participants in the IG reported a reduced level of distress compared to participants in the CG in the ITT population (*F*_1,215_=4.7; *P*=.03; ηp²=0.02; Table 3). The observed treatment effect was more pronounced in the PP population (*F*_1,121_=6.9; *P*=.01; ηp²=0.05). The analysis revealed that higher levels of baseline distress predicted a greater change in distress after 12 weeks in the IG. An exploratory regression analysis yielded no predictive effect of age on the change in distress. The explorative sensitivity analysis among all randomized participants (n=248) showed comparable treatment effects (jump-to-control: *F*_1,19375.1_=5.3; *P*=.02; ηp²=0.02 and jump-to-intervention: *F*_1,15314.8_=5.9; *P*=.02; ηp²=0.02).

**Table 3:** Primary outcome: change in distress between baseline and follow-up (12 weeks)^a^.

			Intervention^b^					Control^c^					Analysis of covariance				
			Values, n		Values, mean (SD)		Values, n			Values, mean (SD)		*F* test (*df*)			*P* value		ηp²
**Intention-to-treat**																	
	Baseline	99		6.0 (2.1)		119			6.0 (2.4)		N/A^d^			N/A		N/A	
	Follow-up	99		5.1 (2.5)		119			5.9 (2.5)		N/A			N/A		N/A	
	Change	99		–0.8 (2.8)		119			–0.1 (2.9)		4.7 (1, 215)			.03		0.02	
**Per-protocol**																	
	Baseline	50		6.0 (2.2)		74			5.7 (2.2)		N/A			N/A		N/A	
	Follow-up	50		4.8 (2.8)		74			5.9 (2.4)		N/A			N/A		N/A	
	Change	50		–1.2 (3.2)		74			0.2 (2.8)		6.9 (1, 121)			.01		0.05	

^a^An analysis of covariance was used to test for differences in change in distress levels between groups from baseline to follow-up (12 weeks), controlling for baseline. The partial eta–squared is the reported standardized effect size for the mean difference. The effect sizes can be interpreted as small, medium, or large at ≥0.01, ≥0.06, and ≥0.14, respectively. The results of the intention-to-treat and per-protocol analysis are reported.

^b^Intervention=12-week access to digital therapeutic app intervention+usual care.

^c^Control=usual care.

^d^N/A: not applicable.

### Secondary Outcomes

In the ITT population, symptoms of anxiety (*F*_1,215_=4.8; *P*=.03; ηp²=0.02), depression (*F*_1,215_=15.5; *P*<.001; ηp²=0.07), and fatigue (*F*_1,215_=4.4; *P*=.04; ηp²=0.02) improved in participants in the IG from baseline to 12 weeks compared to participants in the CG (Table 4). The observed treatment effects on anxiety and depression were more pronounced in the PP population (anxiety: *F*_1,121_=7.2; *P*=.01; ηp²=0.06 and depression: *F*_1,121_=14.9; *P*<.001; ηp²=0.11). A trend-to-significant treatment effect was observed for fatigue symptoms in the PP population (*F*_1,121_=3.8; *P*=.05; ηp²=0.03). QoL did not differ significantly between the groups at 12 weeks (ITT: t_216_=0.88; *P*=.38; g=0.12 and PP: t_122_=1.63; *P*=.11; g=0.30).

**Table 4:** Secondary outcomes: changes in anxiety and depression symptoms and fatigue between baseline and follow-up (12 weeks) and quality of life (QoL) at follow-up (12 weeks).

				Intervention^a^				Control^b^					Analysis of covariance								
				Values, n		Values, mean (SD)		Values, n		Values, mean (SD)		*F* test (*df*)			*t* test (*df*)		*P* value		ηp^2^		Hedges *g*
**Anxiety (HADS-A^c^)**																					
	**ITT^d^**																				
		Baseline	99		8.6 (4.4)		119		8.2 (3.9)		N/A^e^			N/A		N/A		N/A		N/A	
		Follow-up	99		7.7 (4.3)		119		8.2 (4.1)		N/A			N/A		N/A		N/A		N/A	
		Change	99		–0.9 (2.6)		119		0.0 (3.0)		4.8 (1, 215)			N/A		*.03* ^f^		0.02		N/A	
	**PP^g^**																				
		Baseline	50		8.0 (4.4)		74		7.6 (3.6)		N/A			N/A		N/A		N/A		N/A	
		Follow-up	50		7.0 (4.3)		74		7.8 (4.0)		N/A			N/A		N/A		N/A		N/A	
		Change	50		–1.0 (2.6)		74		0.2 (2.3)		7.2 (1, 121)			N/A		*.01*		0.06		N/A	
**Depression (HADS-D^h^)**																					
	**ITT**																				
		Baseline	99		7.2 (4.4)		119		6.6 (4.1)		N/A			N/A		N/A		N/A		N/A	
		Follow-up	99		6.4 (4.5)		119		7.5 (4.9)		N/A			N/A		N/A		N/A		N/A	
		Change	99		–0.8 (2.8)		119		0.9 (3.5)		15.5 (1, 215)			N/A		*<.001*		0.07		N/A	
	**PP**																				
		Baseline	50		6.7 (4.3)		74		6.1 (3.7)		N/A			N/A		N/A		N/A		N/A	
		Follow-up	50		5.6 (4.4)		74		7.1 (4.7)		N/A			N/A		N/A		N/A		N/A	
		Change	50		–1.1 (3.0)		74		1.1 (3.0)		14.9 (1, 121)			N/A		*<.001*		0.11		N/A	
**Fatigue (FACIT-F^i^)**																					
	**ITT**																				
		Baseline	99		29.2 (10.6)		119		31.1 (11.4)		N/A			N/A		N/A		N/A		N/A	
		Follow-up	99		32.2 (10.8)		119		31.2 (13.0)		N/A			N/A		N/A		N/A		N/A	
		Change	99		3.0 (8.6)		119		0.1 (9.3)		4.4 (1, 215)			N/A		*.04*		0.02		N/A	
	**PP**																				
		Baseline	50		30.1 (11.5)		74		32.1 (11.2)		N/A			N/A		N/A		N/A		N/A	
		Follow-up	50		34.2 (11.4)		74		32.9 (12.8)		N/A			N/A		N/A		N/A		N/A	
		Change	50		4.2 (8.8)		74		0.8 (8.3)		3.8 (1, 121)			N/A		.05		0.03		N/A	
**Improvement in QoL (CGI-I^j^)**																					
	**ITT**																				
		Follow-up	99		3.8 (1.1)		119		3.9 (1.4)		N/A			0.88 (216)		.38		N/A		0.12	
	**PP**																				
		Follow-up	50		3.7 (1.2)		74		4.1 (1.3)		N/A			1.63 (122)		.11		N/A		0.30	

^a^Intervention=12-week access to digital therapeutic app intervention+usual care.

^b^Control=usual care.

^c^HADS-A: Hospital Anxiety and Depression Scale, anxiety subscale (German version, at baseline, cutoff score ≥8).

^d^ITT: intention-to-treat.

^e^N/A: not applicable.

^f^Italicized values are significant at *P*<.05.

^g^PP: per-protocol.

^h^HADS-D: Hospital Anxiety and Depression Scale, depression subscale (German version, at baseline, cutoff score ≥8).

^i^FACIT-F Functional Assessment of Chronic Illness Therapy–Fatigue.

^j^CGI-I: Clinical Global Impression Improvement.

### Safety Outcomes

IG participants reported no adverse reactions or side effects of digital therapeutic during the study.

### Intervention Adherence and Engagement

Of the 99 participants in the IG (ITT), 98 (99%), 78 (79%), and 67 (68%) used the digital therapeutic intervention at 0- to 4-, 5- to 8-, and 9- to 12-week periods of the 12-week intervention, respectively, demonstrating good initial adherence to the intervention, which decreased moderately over time. App use (module use and days spent on the app) decreased over time (Table 5). IG participants accessed content from various categories at different frequencies. The most accessed categories were cancer therapy, symptoms and side effects, and nutrition in cancer, with 80% (79/99), 83% (82/99), and 80% (79/99) of users accessing the content in these categories, respectively. Conversely, partnership and family, relaxation, and recipes were accessed less, with 29% (28/99), 34% (33/99), and 32% (31/99) of users, respectively.

**Table 5:** App use in the intervention group over time (n=99).

	Week 0-4 (n=98)	Week 5-8 (n=78)	Week 9-12 (n=67)
Days of app use, median (IQR)	9 (4-15)	3 (2-14)	4 (2-10)
Tracking of distress in days, median (IQR)	5 (1-12)	2 (0-14)	1 (0-6)
Articles read, median (IQR)	6 (2-16)	2 (0-9)	1 (0-5)
Patients using a Journey, n (%)	64 (65.3)	18 (23.1)	12 (17.9)

## Discussion

### Principal Findings

This nationwide waitlist RCT examined the efficacy of Mika, an app-based digital therapeutic that provides a personalized supportive intervention for patients with cancer. Participants who had access to the Mika app for 12 weeks showed significant improvements in perceived distress (ie, the primary outcome) and symptoms of anxiety, depression, and fatigue (ie, the secondary outcomes) compared to participants who received UC. The observed treatment effects were similar in the ITT and PP populations but more pronounced in the PP population, indicating the overall robustness of the findings. We observed no group difference in the QoL after 12 weeks. Intervention adherence was good, and no adverse reactions or side effects of the investigated digital therapeutic were reported.

### Comparison With Prior Work

While a growing body of research shows evidence of the efficacy of app-based interventions for oncological populations on distress, fatigue, anxiety, and depression [20,25,27,31,51], this is the first study to examine the efficacy of a single holistic app-based digital therapeutic based on multiple intervention modules on these patient-relevant outcomes. Although the improvement in the primary outcome was modest, it reflects the nuanced nature of psycho-oncological interventions, where even modest changes can have significant clinical relevance. Furthermore, we conducted comprehensive testing of the effects of the investigated digital therapeutic on patients with cancer across all tumor entities, using a larger sample size compared to most previous studies [25,27,31,51].

In contrast to the findings of this study, however, other studies found an effect of app-based supportive interventions on QoL [23,27,31]. This difference in findings could be due to differences in the operationalization and measurement of QoL. In this study, participants’ global QoL was assessed using a single-item questionnaire after a 12-week intervention period. However, global QoL has been shown to be less affected in patients with cancer compared to specific components of QoL, such as social or cognitive functioning and symptom burden from fatigue or insomnia [52]. Further research using different QoL assessment tools could provide more insights into the efficacy of the investigated digital therapeutic on specific aspects of QoL.

A significant level of intervention adherence and engagement with the digital therapeutic, with varying degrees of interaction across the different app modules, indicates good acceptability and perceived subjective benefit of the investigated digital therapeutic and allows for reliable conclusions about its efficacy in oncological settings. The broad range of engagement, as illustrated by the IQRs, underscores the personalized nature of app use, catering to diverse participant needs and preferences. The variability in engagement levels across different app modules highlights the importance of personalizing digital therapeutics to increase adherence and maximize therapeutic effects.

As we evaluated the app intervention holistically, future studies should examine the impact of the app’s individual components.

While the dropout rate in the IG was slightly higher than that in the CG, the dropout rate in the IG as well as the overall dropout rate was low compared to other app-based supportive interventions [25,30]. Considering that patients with cancer have been found to have a positive attitude toward digital health [53,54], the findings of this study add to the notion that digital health interventions have the potential to overcome barriers associated with access to supportive care in oncological populations [55].

We found a positive effect of the investigated digital therapeutic on general psychological distress and a broad range of specific distress-associated parameters. Importantly, improvements in psychological symptoms, that is, depression and anxiety, can also have a positive tertiary preventive effect on cancer progression [5]. The effect sizes in this study ranged from small (ηp²=0.02) to medium (ηp²=0.07) in the ITT population and were more pronounced in the PP population (ηp²=0.05-0.11). The primary outcome improvement, while subtle, aligns with the expected outcomes in psycho-oncological interventions, highlighting the importance of considering the broad spectrum of therapeutic impacts. The medium to large effects observed in secondary end points, together with the primary outcome, illustrate the broad therapeutic impact and highlight the digital therapeutic’s capacity to significantly improve key aspects of psychological well-being in patients with cancer. Small-to-medium effect sizes are common in in-person supportive care interventions [6]. Our results also compare well with other app-based supportive care interventions, such as small effect sizes reported for a CBT and psychoeducation self-management apps on fatigue [25] or small to medium effects of a web-based mindfulness-based intervention on anxiety and depression [56]. This is further supported by the results of several systematic reviews [20,21]. The fact that such effect sizes can be achieved with minimal cost and personnel effort via a digital approach further supports the significant potential for accessibility, reach, and impact of digital therapeutics.

### Clinical Implications

The multifaceted intervention modules of the investigated digital therapeutic aim to support patients holistically. The investigated digital therapeutic hereby translates widely used evidence-based intervention methods within supportive care, such as symptom monitoring; patient education; modules of CBT, MBSR, and acceptance and commitment therapy; and strength and flexibility training, into a digital format. The intervention modules of the app are designed to help patients learn about their disease and prepare for discussions with clinicians in an informed decision-making process. This may reduce anxiety and insecurities across the cancer trajectory, while empowering patients and strengthening their self-efficacy.

While it is acknowledged that digital therapeutic interventions might not fully replicate the “in-person” experience, the scope and utility of these tools in the realm of oncology are substantial. For instance, a study evaluating a mobile app designed for tracking patient-reported daily activities found that when supervised by a physician, the data collected were more accurate than when used without guidance [57]. Conversely, a music app was equally effective in alleviating pain and anxiety in emergency department patients irrespective of supervision [58]. This suggests that certain interventions, such as symptom tracking, might be more prone to inaccuracies without proper guidance than passive activities, such as listening to music. In addition, CBT, which is traditionally the most effective in face-to-face settings, has generated interest in the digital domain. A study on the digital adaptation of mindfulness-based cognitive therapy for patients with cancer experiencing distress found the therapeutic connection between therapist and patient to be as potent as in in-person sessions [59]. This underlines the evolving role of digital therapeutics and its potential to reshape therapeutic avenues in oncology, thus paving the way for enhanced patient care.

Furthermore, considering the increasing number of patients with cancer experiencing psychosocial distress and the limited availability of health care professionals, digital therapeutics could present scalable and cost-effective solutions. These solutions can address symptoms and bolster the quality and accessibility of supportive care [55,60,61]. Recognizing patients’ diverse needs, tools such as the Mika app leverage artificial intelligence to deliver real-time, tailored support. This has the potential to benefit a broad spectrum of patients with cancer globally while also reducing the pressure on health care infrastructure and professionals. Therefore, digital therapeutics offer a patient-focused approach that is adaptable to specific clinical and lifestyle challenges such as disease management, emotional support, and health-related determinants. They might also further enhance medication adherence, tolerance to chemotherapy, and overall survival rate in the cancer care continuum [15]. Incorporating these digital tools into routine oncological supportive care can augment patient-centric care and enrich patient experience, safety, and interactions with clinicians [15,61]. However, while there is a consensus among medical professionals and stakeholders regarding the revolutionary potential of digital health in addressing cancer treatment challenges, the path to universal adoption remains intricate. Future studies should delve into the assimilation of digital therapeutics, such as Mika, into standard care across varied clinical environments and evaluate hurdles such as digital literacy and the acceptance of digital tools by both patients and health care professionals [62-64].

### Strengths and Limitations

The main strength of this study was the app itself. It addresses the overreaching problem areas faced by all patients with cancer while providing tailored support for population-specific areas of burden (ie, cancer type, treatment status, and use behavior). Its flexible and easily accessible use allows for seamless integration into patients’ daily lives and continuity of supportive treatment. In addition, the low overall dropout rate and data monitoring led to very little missing data. Similar findings in the ITT, PP, and extended ITT populations suggest overall robustness of the results.

This study has several limitations. First, the web-based recruitment procedure may have led to study registration from patients with cancer who were particularly motivated, digitally literate, and highly functioning in seeking support during their cancer journey, which may limit the generalizability of the study. However, the use of additional recruitment pathways (support groups and participant pool) likely resulted in the recruitment of a more heterogeneous sample, possibly compensating for potential selection bias. Future studies might investigate the impact of various recruitment channels on the efficacy of digital therapeutics, and thus, which population may be particularly responsive to digital interventions. Second, the higher number of dropouts in the IG compared to the CG may reflect treatment dissatisfaction or lost interest in the treatment of some participants, potentially confounding the study’s results. Dropouts, who are more likely to show elevated levels of distress, may have been made aware of the increased need for support through the intervention modules. Patients with clinically significant levels of distress or mental disorders might have accessed support services with more guidance from a health care professional, such as psychotherapy or psycho-oncological counseling. However, no side effects or adverse events were reported in the IG, and the overall robust pattern of results in the ITT, PP, and extended ITT populations suggests a low risk of attrition bias. The fact that participants who dropped out of the study showed higher baseline distress levels may have led to an underestimation of the intervention effect as higher baseline distress levels predicted a greater change in outcome after treatment. Third, due to the nature of the intervention, the group allocation could not be blinded. While experimenter bias was reduced due to a predefined, standardized monitoring procedure and statistical analysis plan, IG participants may have anticipated potential effects. Fourth, the intervention, along with its adherence, was assessed as a whole, which requires the evaluation of specific modules and any potential dose-response relationship in the future. In addition, there was no specific measure to evaluate the subjective usefulness or satisfaction with the digital therapeutic under investigation. Incorporating such a measure could have provided targeted insights into the participants’ perceptions and experiences with the app. However, the observed use behavior, characterized by participants repeatedly accessing the app and actively engaging with its content, may serve as an indirect indicator of the app’s value to the participants. Future studies should aim to validate this interpretation. Finally, the study sample included participants with a wide variety of cancer diagnoses, which did not allow for the examination of diagnosis-specific intervention effects. However, the sample composition is consistent with the target population of the investigated digital therapeutic, which includes patients with cancer of all entities, and strengthens the study’s generalizability and clinical utility. Moreover, a large body of data shows that while variables such as cancer type, treatment status, disease progression, and sex may influence the magnitude of treatment response to supportive therapy, the beneficial effects of supportive therapy are present across various cancer subpopulations [65-67]. In addition, there is a consensus that psychosocial support needs to be integrated into routine cancer care for all cancer types [68,69].

### Conclusions

In summary, this RCT demonstrated that Mika, an app-based digital therapeutic that provides a personalized supportive care intervention, can effectively reduce psychological distress and further alleviate symptoms of anxiety, depression, and fatigue in patients with cancer. Digital therapeutics, such as Mika, deliver easily accessible, patient-centered, and effective psychosocial and self-management support for patients with cancer across the course of the disease. Digital therapeutics may present scalable solutions to support patients with cancer worldwide and thus help fill the supportive care gap. Further research is needed to explore the integration of Mika into routine cancer care and its efficacy in diverse clinical settings.

## Appendix

Multimedia Appendix 1 CONSORT-eHEALTH checklist (V 1.6.1).

## Abbreviations

CBT: cognitive behavioral therapy
CG: control group
IG: intervention group
ITT: intention-to-treat
MBSR: mindfulness-based stress reduction
PP: per-protocol
QoL: quality of life
RCT: randomized controlled trial
UC: usual care

## Data Availability

The data set generated during and analyzed during this study, including individual participant data that underlie the results reported in this article after deidentification (text, tables, and figures), clinical study report, informed consent form, and analytic code, are available from AMT beginning 3 months and ending 5 years following article publication. Access to the data will be granted to investigators whose proposed use of the data has been approved by an independent review committee identified for this purpose, for individual patient data meta-analysis. Proposals for accessing the data may be submitted up to 36 months following article publication.
